# Supplementary material for: Polyketide synthase gene clusters in the ericoid mycorrhizal fungus Oidiodendron maius: genome-wide prediction, expression profiling, and functional characterization of knockout mutants
Source: Front Plant Sci. 2026 Sep 16;17:1924430. doi: 10.3389/fpls.2026.1924430 (PMC13623903; doi:10.3389/fpls.2026.1924430)
Supplement: Supplementary file 1 [file SupplementaryFile1.zip › Supplementary Materials/Supplementary Folder 1.zip!/Oidma1_AssemblyScaffolds_Repeatmasked/knownclusterblast/cluster31/jgi.p_Oidma1_44092_mibig_hits.html]

| MIBiG Protein | Description | MIBiG Cluster | MiBiG Product | % ID | % Coverage | BLAST Score | E-value |
| --- | --- | --- | --- | --- | --- | --- | --- |
| CAP96439.1 |  | BGC0000420 | Roquefortine\_C\_/\_meleagrin\_biosynthetic\_gene\_cluster | 31 | 88.7884267631 | 267 | 4.6e-71 |
| ADY16699.1 | TqaJ | BGC0001142 | Tryptoquialanine\_biosynthetic\_gene\_cluster | 30 | 89.1500904159 | 262 | 1.1e-69 |
| AAS90069.1 | AflT | BGC0000010 | Aflatoxin\_biosynthetic\_gene\_cluster | 30 | 88.7884267631 | 260 | 5.7e-69 |
| AAS89998.1 | AflT | BGC0000007 | Aflatoxin\_biosynthetic\_gene\_cluster | 30 | 88.7884267631 | 259 | 7.4e-69 |
| AAS90046.1 | AflT | BGC0000009 | Aflatoxin\_biosynthetic\_gene\_cluster | 30 | 88.7884267631 | 258 | 1.6e-68 |
| AAS90092.1 | AflT | BGC0000006 | Aflatoxin\_biosynthetic\_gene\_cluster | 30 | 88.7884267631 | 255 | 1.1e-67 |
| AAS90021.1 | AflT | BGC0000008 | Aflatoxin\_biosynthetic\_gene\_cluster | 30 | 88.7884267631 | 255 | 1.4e-67 |
| ACD39756.1 | major\_facilitator\_superfamily\_transporter | BGC0000076 | Hypothemycin\_biosynthetic\_gene\_cluster | 32 | 88.4267631103 | 250 | 5.9e-66 |
| ACD39765.1 | major\_facilitator\_superfamily\_transporter | BGC0000077 | Hypothemycin\_biosynthetic\_gene\_cluster | 32 | 88.4267631103 | 250 | 5.9e-66 |
| BAE71313.1 | putative\_ABC\_transporter | BGC0000004 | Aflatoxin\_biosynthetic\_gene\_cluster | 31 | 78.842676311 | 245 | 1.9e-64 |
| AMR44276.1 | MFS\_multidrug\_transporter | BGC0001398\_c3 | Phomopsins\_biosynthetic\_gene\_cluster | 32 | 87.8842676311 | 243 | 7.2e-64 |
| ctg1\_orf0002 |  | BGC0000688 | Copalyl\_diphosphate\_biosynthetic\_gene\_cluster | 32 | 65.4611211573 | 161 | 3.6e-39 |
| EAA65599.1 | hypothetical\_protein | BGC0000022 | Asperfuranone\_biosynthetic\_gene\_cluster | 27 | 96.9258589512 | 158 | 2.3e-38 |
